# Collaborative virtual reality environment in disaster medicine: moving from single player to multiple learners

**DOI:** 10.1186/s12909-024-05429-8

**Published:** 2024-04-19

**Authors:** Laure Abensur Vuillaume, Jonathan Goffoy, Nadège Dubois, Nathacha Almoyner, Cécile Bardet, Evelyne Dubreucq, Sophie Klenkenberg, Anne-Françoise Donneau, Camille Dib, Alexandre Ghuysen, On Behalf Societe Francophone de Pédagogie Innovante en Santé and Medical Simulation Center of ULiège

**Affiliations:** 1 SAMU 57, Service d’Accueil Des Urgences, CHR Metz-Thionville, Metz, France; 2 Medical Simulation Center, Department of Public Health, Liège University, Liege, Belgium; 3 Service d’Accueil Des Urgences, Hôpital Saint Louis, La Rochelle, France; 4 Service d’Accueil Des Urgences, Centre Hospitalier de Niort, Niort, France; 5 Service d’Accueil Des Urgences, Hôpital TENON, APHP, Paris, France; 6 Biostatistics and Research Methods Center (B-STAT), Liège University, Liege, Belgium

**Keywords:** Disaster medicine, Virtual reality, Medical simulation, Emergency medicine

## Abstract

**Background:**

The use of virtual reality (VR) in healthcare education is on the increase. In disaster medicine, it could be a solution to the cost and logistic constraints for a “full-scale” scenarios. However, VR is mainly designed for single players, which is not appropriate for the objectives pursued in disaster medicine. We decided to evaluate the educational value of using individual VR simulation in disaster medicine on a group of learners.

**Methods:**

The VR scenario used was a reproduction of a major train crash, with 21 victims and whose objectives were START triage and first aid techniques. The sessions were carried out in multi-participant groups with different roles (active and immersed with headset, paper triage without headset, and active for communications not immersed in the headset). Their perceived self-efficacy was assessed before (T0), after (T1) and 2 months (T2) after the training. Satisfaction and confidence in learning were also measured.

**Results:**

The median levels of satisfaction and confidence in learning were of 21/25 and 32/40 respectively. Their perceived self-efficacy increased significantly between T0 and T1 (*p* < 0.001), and remained stable until T2. The different roles of participant showed no difference in terms of satisfaction, confidence in learning or changes in perceived self-efficacy. One third of the participants agreed that the number of participants had interfered with their learning. A significant negative correlation (r_S_ = -0.51, *p* = 0.002) was found between satisfaction and the fact of having been hindered by the number of participants. Around 90% of participants found the activity entertaining and found the new technologies appropriate for learning technical skills.

**Conclusions:**

This first experience of VR in a group setting is satisfactory and shows its positive effects. The limitations highlighted here will enable areas of improvement to be identified for the use of VR in disaster medicine, pending the development of multi-player tools. It would now be appropriate to analyse the impact of this type of simulation on learning and its retention over time.

**Supplementary Information:**

The online version contains supplementary material available at 10.1186/s12909-024-05429-8.

## Background

Over the past decades, Virtual Reality (VR) use has moved from gaming to various field such as military and aviation trainings, architectural design and education. Using a virtual reality headset, virtual reality simulation aims to replicate a real-life or health care situation using highly visual, immersive 3D technology [1]. In healthcare, VR was mainly implemented initially for therapeutic purposes, (e.g. managing phobia or anxiety) [2, 3] then in healthcare education (e.g. surgical procedures, team- work) [4–6]. In this field, it appears to be equivalent or superior to traditional teaching methods [7]. Through an unprecedented ability to immerge participants in an almost infinite number of environments, VR has the potential to increase learner motivation and commitment [8], which are major determinants of learning in health education [9]. Indeed, the realistic experience and the resulting sense of presence enables the learners to evolve in a reproducible and safe environment, allowing them the opportunity to learn from potential mistakes and to improve their technical and non-technical skills. Other potential benefits are to be found in the lower costs related to human and material resources [10], the opportunity to duplicate potentially hazardous or complex environments [11], the ethical respect considerations [12], data collection and feedback [13].

Disaster medicine, a specific area of emergency medicine national curricula, remains difficult to teach with physicians and students still having shortcomings in this discipline [14]. Indeed, to improve the effectiveness of disaster medicine education, stressful and realistic environments need to be simulated to teach learners how to apply protocols and algorithms under such constraints [15]. Although interesting, "full-scale" training event require a lot of material and human resources, with questionable cost-effectiveness at the end. Due to their complexity or scarcity, many situations are almost impossible to replicated or lack realism. The creation of varied, multi-modal education programs, notably including VR environment use, might help overcome these difficulties [16]. Several organizations have implemented VR simulations to increase the quality and relevance of disaster medicine teaching [17, 18] with promising results [19].

However, most game-based learnings through VRs are limited to a single learner immersive 3D experience. Apart for the need for large scale training and the number of students to be trained, another specific challenge for disaster medicine [20], is that such training is intrinsically linked to inter-disciplinary, teamwork, leadership or communication between participants. Few multiplayer VRs have been developed to date, with encouraging results in terms of education effectiveness [21, 22]. In addition, there seems to be room for VR experience to be shared by multiple participants through video-display of one particular participants ‘experience to a larger group and their participation in observation and debriefing. Indeed, even in multiplayer VR systems, facilitation and learning is usually performed by a trained instructor, leading the exercise, debriefing the participants’ experience or the peer-to-peer interactions. Disaster preparedness, plan and response to a hypothetical mass casualty incident include several principles such as mass triage, surge capacity, incident command system, coordination, inter-disciplinary communication and mutual support. In such circumstances, several stakeholders, such as the first responders responsible for triage at the scene, might been seen as “single players”, having to share many information through the incident command chain.

Such very specific features of VR gaming learning experience and requirement for disaster medicine training and education led us to wonder about the possibility of extending the educational experience using virtual reality beyond the participant immersed in the scene of intervention to the whole group, via the assignment of these group members to tasks related to the general organization of care in a disaster situation.

Wishing to use the available resources, we tested the use of a VR environment initially intended for a single learner within a group of learners. The main objective of this study was to evaluate the educational value of using individual VR simulation in disaster medicine on a group of learners.

## Material and methods

### Study setting and design

The VR scenario was based on a major train crash with multiple victims, designed and validated by a team of experts (nurses, emergency physicians) [6, 23, 24] to train the participant apply START triage and first aid techniques (tourniquet placement, bleeding management, etc.) in specific situations. The VR scenario was initially created for a single player and is regularly used at the Liège University medical simulation center.

This scenario was presented during a workshop at the French Society of Emergency Medicine conference in Poitiers in October 2022, with the support of the Société Francophone de Pédagogie Innovante en Santé and the Medical simulation Center of the University of Liège. To adapt the Belgian codes to the French disaster medicine codes and adapt the debriefing, two French experts in emergency and disaster medicine composed this working group (N.A., and CB).

To adapt the single –player game to multiple players, we brought together all the experts for disaster medicine, simulation and gamification (LAV, N.A, CB, JG, ND) to consider the position and role of each participant.

The primary objective was to assess the impact of our simulation session on learner satisfaction and confidence. The secondary objective is to evaluate the feasibility of multi-player learning using a single-player VR device.

### Study population

We included all the participants who enrolled in one of the 4 VR training workshop on triage in emergency situations at the French Society of Emergency Medicine conference in Poitiers in October 2022 (*n* = 37). This was a voluntary, non-probability sample. The participants signed a voluntary and informed consent form and were informed in writing and orally.

### Data collection

We used several tools for data gathering:

- *Sociodemographic questionnaire* exploring gender, previous training and participation type (supplemental material).
- *Learner satisfaction scale* 17: This scale is divided into two parts: satisfaction with learning (ESA, with a score ranging from 5 to 25) and in self- learning confidence (ECEA, with a score ranging from 8 to 40). Several statements assess these two parts of the questionnaire. Learners were asked to score from 1 (totally disagree with the statement) to 5 (totally agree with the statement) (supplemental material).
- *Satisfaction list of statements*: This non-validated questionnaire contained statements that learners were asked to rate from "Totally disagree" to "Totally agree" with the statement. One is completed just after the intervention, the other 2 months after (supplemental material).
- *Perceived self-efficacy*: Self-evaluation questions regarding the triage skill composed of 3 areas: knowledge about the skill; ability to manage the skill; and applying the skill in practice. Students self-evaluated them using a Likert scale ranging from 0 (not at all) to 5 (entirely).

All data were collected in pseudonymized form, analyzed and stored in anonymized form.

### Intervention

The simulation session was performed according to the basic principles of a simulation session 15, starting with a pre-briefing phase and VR equipment familiarization (± 10 min), followed by the simulation exercise (± 35 to 40 min per group) and finally the debriefing (15 min). Debriefing tracks had been established beforehand to meet the educational objectives set. One session could accommodate a maximum of 10 participants.

**Fig. 1:**
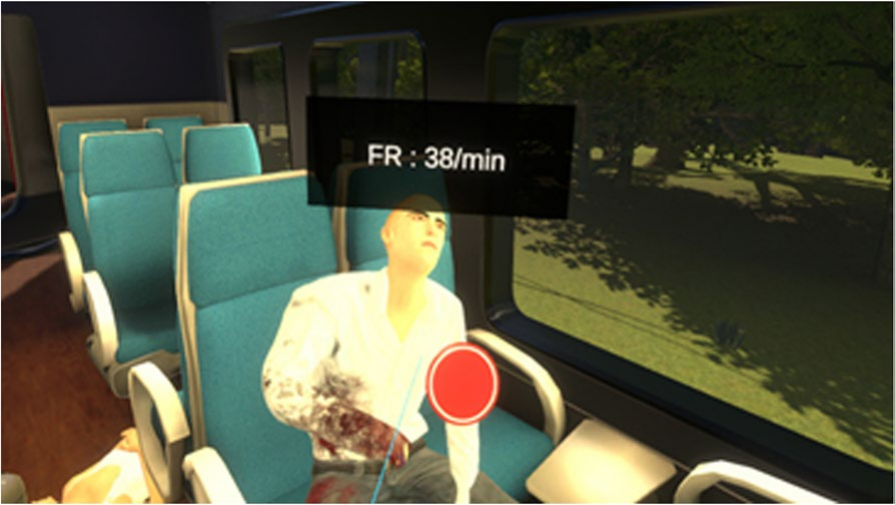
Example of a victim in the first carriage. Traumatic amputation of the right hand with hemorrhage. Respiratory rate is 38/min

In the virtual environment of the train crash, the player has a disaster bag containing coloured badges for triage, tourniquets, dressings and 3-sided dressings. The player can enter the first 3 secure carriages where there are victims calling for help. Using the controller, the player can obtain information about the victim's state of consciousness, respiratory rate and heart rate, as well as whether or not they can move. The 21 victims present a range of clinical situations, from psychological shock to traumatic hand amputation or death. It is possible to get close, examine the victims' wounds and even remove their clothing if necessary (Fig. 1).

Accordingly, six participants were active during the simulation.All participants had access to the same data at the same time. The simulation scenario assigned the following roles to the participants, on a voluntary basis:

- 4 Single players (These four players wore headset in succession): 1 person who made a quick initial pass through the 3 carriages, 3 people who each triaged 1 carriage and made an oral report to the medical director
- 1 medical director (without the headset, who received and gave information orally)
- 1 reporter who made a final oral assessment (without the headset)

**Fig. 2:**
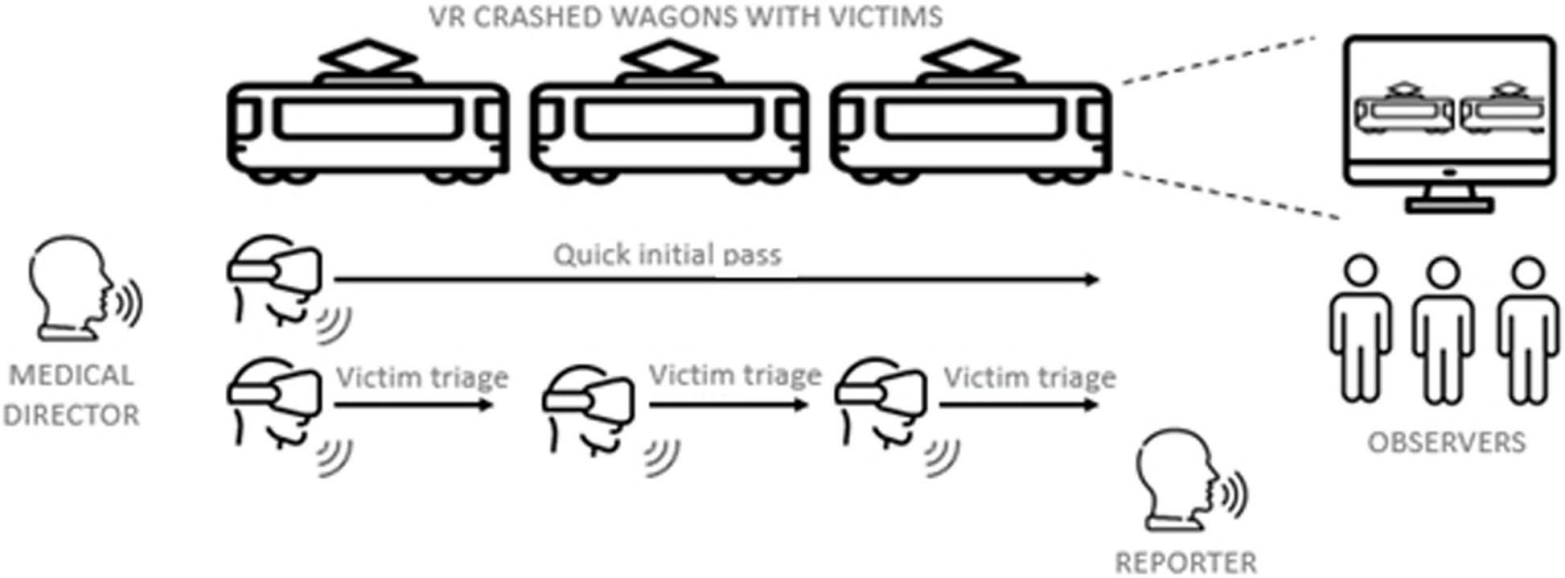
Multiplayer simulation sequence

The remaining 3 or 4 participants were considered as “observers” and had to carry out a triage "on paper" by observing the simulation and the parameters of the victims via a display on a large screen (Fig. 2). On the paper documents, the observers found a photo of each victim to perform the START sorting. They observed the characteristics of the victims with the help of the live retransmission on the large screen, on the basis of the two passages.

Before the simulation (T0), all participants were asked to fulfill an informed consent, a socio-demographic questionnaire and the self-efficacy questionnaire. After the debriefing (T1): participants received the satisfaction and confidence questionnaire as well as the self-efficacy questionnaire. Finally, participants were contacted 2 to 3 months after the training to answer a satisfaction questionnaire online and also the self-efficacy one (T2).

### Statistical analysis

Qualitative variables were described using frequency tables, while ordinal variables were summarized using medians and interquartile ranges (IQR), as well as frequency tables when the number of modalities was not too large.

Comparisons of questions between different groups were realized using non-parametric Kruskal–Wallis tests, and analysis of changes between different timepoints were assessed using non-parametric Friedman tests. In case of significant differences, post-hoc tests with Benjamini & Yekutieli correction were performed. Association between ordinal variables were investigated using Spearman correlation coefficients.

Analyses were always performed on the maximum amount of available data, and missing values were not replaced. Statistical significance was achieved at 95% confidence (*p*-value significance < 0.05).

Analyses were performed using R software, version 4.1.1.

## Results

### Participant characteristics

Half of the participants took an active part (with headset) in the training, while a quarter of the participants were "paper" participants (without headset) and the others were active without headset (oral reports and transmissions). However, this information was only available for 25 of the 37 participants, i.e. 68% of them. Twelve participants didn’t communicate about their role in the simulation (missing data). Almost none of the participants (*n* = 3, 8.33%) had previous training in virtual reality, while almost three quarters had previous training in simulation (*n* = 26, 72.2%). Finally, over half had already used virtual reality as a hobby (Table 1).

**Table 1:** Descriptive statistics of sociodemographic characteristics (*n* = 37)

Parameters	n	Respondents (%)
Gender	37	
Female		19 (51.35)
Male		18 (48.65)
Type of participation	25	
Observers = observers, triage on paper without headset		7 (28.00)
Active with headset		12 (48.00)
Paper = Active without headset (oral communication)		6 (24.00)
Previous training in virtual reality	36	
No		33 (91.67)
Yes		3 (8.33)
Previous training in simulation	36	
No		10 (27.78)
Yes		26 (72.22)
Previous use virtual simulation as a hobby	36	
No		20 (55.56)
Yes		16 (44.44)

### Learners satisfaction

Regarding the overall satisfaction assessment (Table 2), 90.0% of participants agreeing or totally agreeing with the statements related to the activity being entertaining, new technologies being appropriate for learning technical skills and agreement to recommend the learning activity to a colleague. It should be noted that the numbers of "undecided" participants for the other items were not negligible. Lastly, the statements about the positive impact of the number of participants on learning and the quality of the virtual environment reached an agreement for 33.3% and 58.3% of participants respectively.

**Table 2:** Descriptive statistics for items relating to overall satisfaction after training (T1)

Parameter	n	Median (IQR)	Totally disagree	Disagree	Indecisive	Agree	Totally agree
Motivation	35	4.0 (4.0—5.0)	0 (0.00)	1 (2.86)	7 (20.00)	14 (40.00)	13 (37.14)
Entertaining	37	5.0 (4.0—5.0)	1 (2.70)	0 (0.00)	1 (2.70)	13 (35.14)	22 (59.46)
Technologies for technical skills	36	4.5 (4.0—5.0)	1 (2.78)	1 (2.78)	3 (8.33)	13 (36.11)	18 (50.00)
Technologies for non-technical skills	37	4.0 (3.0—5.0)	0 (0.00)	1 (2.70)	12 (32.43)	14 (37.84)	10 (27.03)
Advice	37	4.0 (4.0—5.0)	1 (2.70)	0 (0.00)	3 (8.11)	18 (48.65)	15 (40.54)
Other teaching method	34	2.0 (2.0—3.0)	7 (20.59)	11 (32.35)	9 (26.47)	6 (17.65)	1 (2.94)
Inconvenient participants' number	36	2.5 (1.0—4.0)	11 (30.56)	7 (19.44)	6 (16.67)	10 (27.78)	2 (5.56)
Appropriate participants' number	36	3.0 (2.0—4.0)	6 (16.67)	7 (19.44)	11 (30.56)	8 (22.22)	4 (11.11)
Realistic	37	4.0 (4.0—4.0)	2 (5.41)	1 (2.70)	5 (13.51)	20 (54.05)	9 (24.32)
Quality	36	4.0 (3.0—4.0)	3 (8.33)	4 (11.11)	8 (22.22)	14 (38.89)	7 (19.44)

Full description of parameters in the annexes

Full description of parameters in the Annexe 1: Satisfaction list of statements (T1)

Participants answered open-ended questions about the strengths and weaknesses of the activity, and the answers corroborated the above results. As for the strong points, the entertaining aspect and the generated motivation were cited 16 times. Realism and a sense of immersion were also mentioned 15 times. In terms of weaknesses, the most frequently cited themes were the complexity of handling the equipment, the limitations and constraints of the scenario, and the fact that it was too short or involved too many participants, with 18, 11 and 10 mentions respectively.

The participants' satisfaction and confidence in learning levels immediately after the training (T1) were moderately high, with a median of 21.0 (19.0—23.0) for satisfaction (ESA) and 32 (30.0—34.0) for confidence respectively (ECEA) (Table 3).

**Table 3:** Satisfaction and confidence level of the participants in their learning

Parameters	n		Median (IQR)	
***Descriptive statistics of satisfaction and confidence (T1)***				
Satisfaction with learning (from 5 to 25) (ESA)	37		21.0 (19.0—23.0)	
Confidence in my learning (from 8 to 40) (ECEA)	33		32.0 (30.0—34.0)	
***Descriptive statistics of satisfaction two months after training (T2)***				
Satisfaction with learning (from 5 to 25)	12		20.0 (15.0—22.0)	
**Evolution of satisfaction with learning between T1 et T2 (*n* = 12)**				
**Parameter**	**T1**	**T2**	**Difference**	***p*-value**
Satisfaction with learning	20.0 (19.5—22.5)	20.0 (15.0—22.0)	-1.0 (-5.0—0.0)	0.146

T1 (just after training) and T2 (two months after training)

Twelve participants responded to the post 2-month questionnaire. In order to confirm that the results of the analyses carried out on this subset can be extended to all participants, the representativeness of these 12 respondents was assessed by performing Fisher's exact tests on the five socio-demographic variables presented in Table 1, between all 37 participants and this sample. No significant differences emerged from these analyses. Participants' satisfaction with their learning two months after the course had a median of 20.0 (15.0–22.0) (Table 3).

Changes in learning satisfaction among these 12 participants are summarized in Table 3. There was no significant difference between satisfaction expressed just after the course and two months after (*p*-value = 0.146).

Two months after the training, 58.33% of respondents felt that the training had met their expectations, and a large proportion of them (83.34%) said that the training had motivated them to learn more about the subject. In addition, ten of the twelve respondents (83.34%) would willingly take part in a group VR workshops again. On the other hand, only 3 respondents (25%) considered that the training has or will have a significant impact on their practices. Finally, when asked about the number of participants in the training course, ten out of twelve respondents (83.33%) felt that the number of participants was not appropriate. Eight of them would have preferred a group of 2 to 5 participants (Table S1 and S2 in suppl).

### Perceived self-efficacy

The 37 respondents' sense of self-efficacy (score between 0 and 15) improved between T0 and T1 (T0: 8 (6–9), T1: 11 (9–12), *p* < 0.05).

**Fig. 3:**
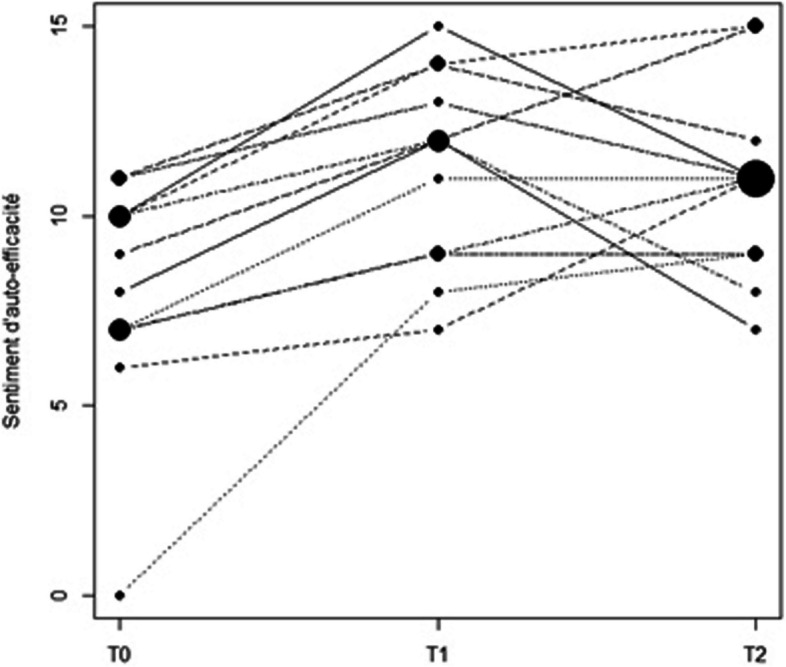
Evolution of self-efficacy score between T0 (before training), T1 (just after training) and T2 (two months after training)

With regard to the 12 participant who responded to the questionnaire sent two months after the training workshop, the levels of self-efficacy are distributed differently at the 3 points time studied (overall *p*-value = 0.002). When we compare the time points two-by-two, we observed a significant positive changes between T0 and T1 and between T0 and T2, with for both a median difference equal to 3.00 (corrected *p*-values equal to 0.005 and 0.025 respectively). However, the median difference between T1 and T2 was found to be zero and not significant (Table 4). The links between the scores of the 12 concerned participants are depicted in Fig. 3.

**Table 4:** Difference between self-efficacy scores before training (T0), just after (T1) and two months after training (T2) for 12 participants

	**T1**	**T2**	**Global *p*-value**
**T0**	3.0 (2.0—4.0)	3.00 (0.5—5.0)	
	**p**_**ex**_** < 0.001**	**p**_**ex**_** = 0.009**	
	**p**_**corr**_** = 0.005**	**p**_**corr**_** = 0.025**	0.002
**T1**		0.00 (-3.0—1.5)	
		p_ex_ = 0.614	
		p_corr_ = 0.999	

T0 (before training), T1 (just after training) and T2 (two months after training)

### Comparisons between types of participation

Evolution in feelings of self-efficacy between T0 and T1, satisfaction with learning and confidence in learning were compared between the three roles of participation in training. There was no significant difference (*p* > 0.05), despite a slightly higher median learning confidence score for active participants (median score equal to 34 (31.5–34.5) compared with 31 (27.0–35.0) for observers, paper and 28.5 (26.0–32.0) for active oral participants).

It should be noted, however, that most of the "active with headset" and "active orally" participants had already taken simulation training beforehand (10 of the 12 active participants and all of the 6 active orally), whereas among the observers/paper, only 3 out of 7 had already taken such training. No difference was also detected for the data 2 months after training. All the results are available in supplementary material (Table S3).

### Perceived impact of the number of participants

Table 5 insights at the links between the fact that the number of participants in the training hindered them (response to the question: "The number of participants in this activity hindered my learning") and the participants' satisfaction, confidence and changes in their perceived self-efficacy. The only significant association was observed to be negative with satisfaction with learning at T1 (r_S_ = -0.51, *p* = 0.002).

**Table 5:** The links between being hindered by the number of participants and satisfaction, confidence in learning and evolutions in feelings of self-efficacy

Parameters	N	Spearman correlation	*p*-value
Satisfaction with learning (T1)	36	*r* = -0.51	**0.002**
Confidence in my learning (T1)	32	*r* = -0.32	0.070
Evolution of self-efficacy score (T0/T1)	36	*r* = -0.26	0.126
Evolution of self-efficacy score (T1/T2)	12	*r* = 0.31	0.331
Evolution of self-efficacy score (T0/T2)	36	*r* = -0.26	0.126

T0 (before training), T1 (just after training) and T2 (two months after training)

## Discussion

To the best of our knowledge, our pilot study is the first to question the possibility of extending the educational impact of using virtual reality by a single player to a broader audience, in the context of disaster medicine training. We found that the participants in our VR group sessions were highly satisfied. In addition, group VR led to an increase in the professionals' sense of self-efficacy, whatever the role of the participants in the simulation. Moving from single player to multiple learners therefore sounds as a very interesting training option. We found that this group effect might have a limit in terms of participant’s number. Indeed, the majority of our respondents felt that the size of the group at our workshop (8 to 10) was unsuitable. They would have preferred groups of 2 to 5 simultaneous participants. We also noticed that there was an inverse relationship between the satisfaction score and the level of agreement that the number of participants might have hindered the learning, suggesting that it would be appropriate to evaluate the ideal number of learners that balance with the ideal learning experience of the participants. In the evaluation of that balance, it should also be noticed that the number of participants was not linked to the perceived confidence in learning or feelings of self-efficacy.

In disaster medicine, *Behmedi *et al. compared the effect of virtual-based medical education versus lecture-based method in teaching start triage lessons in emergency medical students. Mean learning scores of VR simulation-based education were slightly (but not significantly) higher than those of a lecture-based training course, whereas satisfaction was significantly higher in the VR group [25]. *Luigi *et al. also found similar results [26]. These results on satisfaction, which corroborate our own, suggest that the wider use of VR could be beneficial to training in disaster medicine. Some authors in other specialties have been able to show significant results in terms of learning, including for technical procedures, thus encouraging their wider implementation in health education [27–29].

VR has also been demonstrated to enhance the retention of knowledge and triage skills, and can also be used to compare the performance of different triage systems [30]. Like any serious game, it enables learning through error and repetition, by evolving in a safe environment and allowing several cognitive schemas to be tested. McGrath et al., in an expert consensus on the use of VR simulation in the teaching of emergency medicine, also confirmed the need to create targeted environments and advanced technology for teaching and assessment [20]. One of the major technological advances is, in our opinion, the development of multiplayer platforms, enabling a multidisciplinary and interprofessional approach [31, 32]. In particular, they enable team debriefing and the learning and evaluation of team communication. The consensus of McGrath et al. emphasizes the need to develop tools related to these skills [20]. These technological developments must be supported by multidisciplinary teams of doctors, educators, specialists and engineers [33]. Besides, scenario should be developed with to aim to enlarge the potential benefit of learning to a broader audience.

In a complex context, requiring resources and time from both learners and supervisors, VR offers the ability to extend simulation on a larger scale. However, to date most of this VR approach has been developed for a single learner, only partially addressing the problem of resources. To our knowledge, the possibility of teaching a group with single learner tools has not yet been described. Our pilot study therefore provides evidence that needs to be reinforced in order to envisage this possibility and to disseminate VR in teaching environments. This option makes it possible to use VR while awaiting further development of multiplayer environments and the development of virtual teaching platforms as the metaverse promises.

Our pilot study has a number of limitations. First, our study sample was an opportunistic sample, taken at an emergency medicine event. The sample size could therefore have been larger with another approach, although our results do not seem to suffer from a lack of power. Secondly, we did not have a control group. However, the aim of this study was not to compare VR to another group, but rather its ability to be exercised in a group. In addition, the technical constraints of the proposed simulation workshop would not have allowed the control group to be proposed for this event. Finally, it would have been relevant to be able to test several group sizes in order to refine our results, and we encourage further studies on this subject.

## Conclusions

VR is a relevant and interesting tool for teaching disaster medicine, because of its broad technological possibilities and its protective framework, like serious games. Current technological advances do not allow for the widespread use of multiplayer games. Thanks to this pilot study, we were able adapt the simulation scenario on order to test the use of a game initially created for a single player, within a group of learners. Our results indicate that it is possible to use these games for a group of learners, with positive impacts on satisfaction, motivation and learning, as long as the group is not too large and each learner has a pre-defined role in the simulation.

### Supplementary Information

**Supplementary Material 1.**

## Data Availability

The data generated or analyzed during this study are available from the corresponding author on reasonable request.

## Abbreviation

VR: Virtual reality
