# Supplementary material for: Collaborative virtual reality environment in disaster medicine: moving from single player to multiple learners
Source: BMC Med Educ. 2024 Apr 19;24:422. doi: 10.1186/s12909-024-05429-8 (PMC11031920; doi:10.1186/s12909-024-05429-8)
Supplement: Supplementary file 1 — Supplementary Material 1. [file 12909_2024_5429_MOESM1_ESM.docx]

**Annexes : Supplementary material and results**

**Annexe 1: Questionnaires used during the study (in French, translated in English)**

- 1. **Socio-demographic questionnaire**

Sex: male / female

Age :

Status : Doctor / Nurse / Other, please specify :

During the activity I was a :

- An observer
- An 'active' participant - I wore the virtual reality helmet
- An 'active' participant BUT I did NOT wear the virtual reality helmet

In the past, have you taken part in :

Training using virtual reality: YES-NO

Previous training using simulation: YES-NO

Leisure activity using virtual reality: YES-NO

**1.2 Satisfaction with learning questionnaires** (Franklin, 2014, DOI: 10.1016/j.nedt.2014.06.011)

The following statements assess your personal attitudes and feelings about the instruction you receive during the entire training. Each item represents a statement about your attitude towards your self-confidence in the knowledge and skill master. Please indicate to what extent you agree with the following statements from:

1. Very dissatisfied (2) Dissatisfied (3) Neither (4) Satisfied (5) Very satisfied (NA) Not applicable

| **SATISFACTION IN LEARNING (ESA)** | **NA** | **1** | **2** | **3** | **4** | **5** |
| --- | --- | --- | --- | --- | --- | --- |
| The teaching methods used in this simulation were helpful and effective. | □ | □ | □ | □ | □ | □ |
| The simulation provided me with learning materials and activities to promote my learning. | □ | □ | □ | □ | □ | □ |
| I enjoyed how my instructor taught the simulation. | □ | □ | □ | □ | □ | □ |
| The teaching materials used motivating and helped me to learn. | □ | □ | □ | □ | □ | □ |
| The way my instructor taught was suitable to the way I learn. | □ | □ | □ | □ | □ | □ |

| **SELF-CONFIDENCE IN LEARNING (ECEA)** | **NA** | **1** | **2** | **3** | **4** | **5** |
| --- | --- | --- | --- | --- | --- | --- |
| I am confident that I am mastering the content of this training the simulation activity that my instructors presented to me. | □ | □ | □ | □ | □ | □ |
| I am confident that this training simulation covered critical content necessary for the mastery of radiation oncology practice. | □ | □ | □ | □ | □ | □ |
| I am confident that I am developing the skills and obtaining the required knowledge from this training simulation to perform necessary tasks in a clinical setting. | □ | □ | □ | □ | □ | □ |
| It is my responsibility as the student to learn what I need to know from this simulation activity. | □ | □ | □ | □ | □ | □ |
| I know how to get help when I do not understand the concepts covered in the specific simulation or game. | □ | □ | □ | □ | □ | □ |
| I know how to use simulation activities to learn critical aspects of these skills. | □ | □ | □ | □ | □ | □ |
| It is the instructor's responsibility to tell me what I need to learn of the simulation activities content during class time. | □ | □ | □ | □ | □ | □ |

**1.3 Satisfaction list of statements (T1 and T2)**

**Satisfaction and feelings after the workshop using virtual reality.**

**List of statements (T1): This questionnaire contains a list of statements that you must rate from "1 = Totally disagree" to "5 = Totally agree" with the statement.**

| Motivation | Thanks to the use of virtual reality, I was motivated to develop new skills. | 1 | 2 | 3 | 4 | 5 |
| --- | --- | --- | --- | --- | --- | --- |
| Entertaining | I found the activity fun | 1 | 2 | 3 | 4 | 5 |
| Technologies for technical skills | I am convinced that new technologies are well suited to teaching technical skills (sorting victims, placing tourniquets, etc.). | 1 | 2 | 3 | 4 | 5 |
| Technologies for non-technical skills | I'm convinced that new technologies are well-suited to learning non-technical skills (communication skills). | 1 | 2 | 3 | 4 | 5 |
| Advice | I'd recommend this learning activity to a colleague | 1 | 2 | 3 | 4 | 5 |
| Other teaching method | I would have preferred to learn how to sort victims using a different teaching method. | 1 | 2 | 3 | 4 | 5 |
| Inconvenient participants'number | The number of participants in this activity hindered my learning. | 1 | 2 | 3 | 4 | 5 |
| Appropriate participants'number | The number of participants in this activity had a positive impact on my learning. | 1 | 2 | 3 | 4 | 5 |
| Realistic | The proposed scenario was realistic | 1 | 2 | 3 | 4 | 5 |
| Quality | The virtual environment offered was of good quality (image quality, possibilities offered by the game, quality of sound effects). | 1 | 2 | 3 | 4 | 5 |

**List of questions 2 months after the workshop (T2), please answer the following questions.**

| You signed up for a VR workshop on sorting. Did it meet your expectations? | Not at all satisfied | Not satisfied | Neither | Satisfied | Very Satisfied |
| --- | --- | --- | --- | --- | --- |
| Since the workshop in early October, have you had to deal with a mass influx of patients or a disaster? | Yes | No |  |  |  |
| If so, did you use any of the skills acquired during the training? |  |  |  |  |  |
| Since the workshop at the beginning of October, have you used any of the skills or knowledge acquired during the training in another context? | Yes | No |  |  |  |
| Comments, if any |  |  |  |  |  |
| To what extent do you think this training has/will have an impact on your practices? | Not important | Slightly important | Moderately important | Important | Very important |
| I will gladly participate in group VR workshops again. | Strongly disagree | Disagree | Undecided | Agree | Totally agree |
| Did this training motivate me to deepen my knowledge of the subject? | Strongly disagree | Disagree | Undecided | Agree | Totally agree |
| We offered you a training course with 10 participants. In hindsight, do you think this group was appropriate? | Yes | No |  |  |  |
| If not, what would be the ideal number of participants for this type of training? | 1 | 2 to 5 | 6 to 10 | >10 |  |

- 1. **Perceived self-efficacy**

For each objective, estimate your level of knowledge, your ability to act and your motivation to apply this knowledge/skill in your professional activity:

Sorting victims using the START system:

NA - Not applicable

0 - Not at all

1- Very little

2 - A little

3 - A fair amount

4 - Very much

5 - Enormously

|  | NA | 0 | 1 | 2 | 3 | 4 | 5 |
| --- | --- | --- | --- | --- | --- | --- | --- |
| **I know how** to triage and control haemorrhaging in disaster victims. |  |  |  |  |  |  |  |
| **I feel capable** of triaging victims and checking their bleeding during a disaster. |  |  |  |  |  |  |  |
| **I will apply** this knowledge/skill I will apply this knowledge/skill in my professional practice |  |  |  |  |  |  |  |

**Annexe 2: Table S1 - Descriptive statistics for items relating to overall satisfaction 2 months after training (T2)**

| **Table S1 – Statistiques descriptives des items liés à la satisfaction générale après deux mois (T2)** | | |
| --- | --- | --- |
| **Variable** | **Number of respondents** | **Médiane (EIQ) / Number (%)** |
| V_att_post2m | 12 | 4.0 (3.0 – 4.5) |
| Not at all satisfied |  | 0 (0.00) |
| Not very satisfied |  | 2 (16.67) |
| Neutral |  | 3 (25.00) |
| Satisfied |  | 4 (33.33) |
| Very satisfied |  | 3 (25.00) |
| V_cata_post2m | 12 |  |
| No |  | 9 (75.00) |
| Yes |  | 3 (25.00) |
| *V_cata_comp_post2m* | *3* |  |
| *No* |  | *1 (33.33)* |
| *Yes* |  | *2 (66.67)* |
| V_comp_post2m | 12 |  |
| No |  | 8 (66.67) |
| Yes |  | 4 (33.33) |
| V_impact_post2m | 12 | 3.0 (1.5 – 3.5) |
| Not important |  | 3 (25.00) |
| Slightly significant |  | 2 (16.67) |
| Moderately important |  | 4 (33.33) |
| Important |  | 3 (25.00) |
| Very important |  | 0 (0.00) |
| V_part_post2m | 12 | 4.0 (4.0 – 5.0) |
| Totally disagree |  | 0 (0.00) |
| In disagreement |  | 1 (8.33) |
| Undecided |  | 1 (8.33) |
| Agree |  | 5 (41.67) |
| Totally agree |  | 5 (41.67) |
| V_motiv_post2m | 12 | 4.0 (3.0 – 4.0) |
| Totally disagree |  | 0 (0.00) |
| In disagreement |  | 1 (8.33) |
| Undecided |  | 1 (8.33) |
| Agree |  | 5 (41.67) |
| Totally agree |  | 5 (41.67) |

**Annexe 3: Table S2: Descriptive statistics for items relating to the number of training participants (T2)**

| **Table S2: Descriptive statistics for items relating to the number of training participants (T2)** | | |
| --- | --- | --- |
| **Variable** | **Number of respondents** | **N (%)** |
| The number of participants was adapted | 12 |  |
| No |  | 10 (83.33) |
| Yes |  | 2 (16.67) |
| *Ideal number of participants* | *9* |  |
| *Groups of 2 to 5* |  | *8 (88.89)* |
| *Groups of 6 to 10* |  | *1 (11.11)* |

**Annexe 4: Table S3 - Comparison of changes in feelings of self-efficacy between T0 and T1, satisfaction with learning and confidence in learning according to type of participation**

| Variable | Total (n=25) | Observer, Paper (n=7) | Active (with headphones) (n=12) | Orally active (without headphones) (n=6) | p-value |
| --- | --- | --- | --- | --- | --- |
| Difference in sense of self-efficacy (T0/T1) | 3.0 (2.0 - 4.0) | 3.0 (1.0 - 5.0) | 3.0 (2.0 - 4.0) | 2.0 (2.0 - 3.0) | 0.647 |
| Learning satisfaction | 21.0 (19.0 - 23.0) | 20.0 (19.0 - 25.0) | 21.0 (19.5 - 23.5) | 19.5 (19.0 - 20.0) | 0.144 |
| Confidence in learning | 32.0 (27.0 - 34.0) | 31.0 (27.0 - 35.0) | 34.0 (31.5 - 34.5) | 28.5 (26.0 - 32.0) | 0.079 |
